# Genotype-phenotype correlation in multiple endocrine neoplasia type 1

**DOI:** 10.1172/jci.insight.176993

**Published:** 2025-02-13

**Authors:** Charlita C. Worthy, Rana Tora, Chandra N. Uttarkar, James M. Welch, Lynn Bliss, Craig Cochran, Anisha Ninan, Sheila Kumar, Stephen Wank, Sungyoung Auh, Lee S. Weinstein, William F. Simonds, Sunita K. Agarwal, Jenny E. Blau, Smita Jha

**Affiliations:** 1 Metabolic Diseases Branch,; 2 Digestive Diseases Branch, and; 3 Biostatistics Program, National Institute of Diabetes and Digestive and Kidney Diseases, NIH, Bethesda, Maryland, USA.

**Keywords:** Endocrinology, Genetics, Oncology, Calcium, Diagnostic imaging, Molecular diagnosis

## Abstract

**BACKGROUND:**

Among patients with multiple endocrine neoplasia type 1 (MEN1), 80% develop duodenopancreatic neuroendocrine tumors (dpNETs), of whom 15%–25% die of metastasis. There is a need to identify biomarkers to predict aggressive disease. *MEN1* genotype affords an attractive possibility as a biomarker, as it remains constant during life. Currently, patients are clinically diagnosed with MEN1 by the presence of ≥2 primary endocrine tumors (pituitary, parathyroid, and pancreas) or ≥1 primary endocrine tumor with a positive family history. From 10% to 30% of patients diagnosed clinically with MEN1 have no pathogenic germline *MEN1* variants.

**METHODS:**

This was a retrospective study of 162 index patients or probands with genotype-positive and 47 with genotype-negative MEN1 enrolled from 1977 to 2022.

**RESULTS:**

Compared with patients with genotype-negative disease, patients with genotype-positive disease were younger at diagnosis and had an increased frequency of recurrent parathyroid tumors, dpNETs, and angiofibromas or collagenomas. We propose a weighted scoring system to diagnose genotype-positive MEN1 based on clinical characteristics. No evidence of MEN1 mosaicism was seen in 30 tumors from 17 patients with genotype-negative MEN1. Patients with germline MEN1 variants in exons 2 and 3 had a reduced risk of distant metastases.

**CONCLUSION:**

The clinical course of genotype-negative MEN1 is distinct from genotype-positive disease, raising uncertainty about the benefits of lifetime surveillance in patients with genotype-negative disease. MEN1 mosaicism is rare.

**TRIAL REGISTRATION:**

ClinicalTrials.gov NCT04969926

**FUNDING:**

Intramural Research Program of National Institute of Diabetes and Digestive and Kidney Diseases, NIH (ZIA DK043006-46)

## Abstract 2

We confirm that the clinical course of the disease in genotype-negative patients with MEN1 is distinct from those with genotype-positive MEN1 and that these patients may not derive benefit from annual surveillance.

## Introduction

The term “multiple endocrine neoplasia” was coined in the 1900s to describe syndromes with tumors in 2 or more types of hormone-secreting tissues. These were broadly categorized into multiple endocrine neoplasia type 1 (MEN1) and multiple endocrine neoplasia type 2 (MEN2). This report pertains to MEN1, which has a prevalence of 3–20/100,000 and is inherited in an autosomal-dominant pattern with a high degree of penetrance (1). Heterozygous germline inactivating variants in *MEN1* were identified as the main cause of MEN1, some 25 years ago (2, 3). These variants predispose an individual to develop endocrine and nonendocrine tumors after a somatic “second hit” resulting in loss of heterozygosity and biallelic inactivation leading to tumor formation, in accordance with Knudson’s two-hit hypothesis (4). Several studies have shown that patients with MEN1 have a decreased life expectancy with malignant neuroendocrine tumors (NETs) accounting for 50%–70% of the deaths directly related to MEN1 (5–10). While thymic NETs are a rare manifestation in MEN1, duodenopancreatic NETs (dpNETs) have a high prevalence among patients with MEN1 with an age-related penetrance of 80% by the age of 80 (11, 12). However, there is marked heterogeneity in the clinical course of MEN1-related dpNETs, with distant metastases seen in approximately 15%–25% of patients (6, 7, 12–15). Distant metastases correlate negatively with overall survival in patients with MEN1 (6, 16). Thus, most patients with MEN1 have a low risk of distant metastasis from their dpNETs. Moreover, given the heritable nature of the disease and the widespread availability of genetic testing, patients are being increasingly diagnosed with MEN1 even before they develop clinical manifestations. Diagnosis of MEN1 leads to initiation of lifelong serial surveillance scans and laboratory testing, which is currently indicated annually for all patients in the absence of predictors of aggressive disease (12). The identification of predictors of aggressive disease offers the potential to develop a more rigorous surveillance and treatment plan for patients at risk without burdening those who are likely to have an indolent disease course. We postulate that *MEN1* genotype has a role in risk stratification and disease prognostication.

Furthermore, besides a determination based on genetic testing, a diagnosis of MEN1 is made by the presence of at least 2 of the primary MEN1-related manifestations (pituitary adenomas, primary hyperparathyroidism [PHPT], and dpNETs) or at least 1 of the primary MEN1-related manifestations in association with a positive family history of MEN1 in a first-degree relative. Despite the advent of next-generation sequencing, 10%–30% of patients diagnosed with MEN1 based on the aforementioned clinical or familial criteria do not have a germline *MEN1* variant detected on genetic testing (1). Recent studies suggest that the clinical course of MEN1 in genotype-negative patients differs from that in genotype-positive patients (11, 17). However, findings from these studies are limited by inclusion of nonindex patients, small sample size, short follow-up duration, lack of information regarding number of patients who underwent surveillance scans, and lack of sequencing and deletion-duplication analyses not only for *MEN1* but also for additional genes including *CDKN1B* or *CDC73*, which can present with features that overlap and mimic those of MEN1 (18, 19). Thus, there is a need for additional studies of genotype-negative MEN1 to better define the disease-related risk(s) and optimize the surveillance plan for patients with genotype-negative MEN1.

The National Institute of Diabetes and Digestive and Kidney Diseases (NIDDK), NIH, has a long-standing research protocol investigating MEN1 with longitudinal follow-up of patients spanning multiple generations. This research resulted in the identification of germline variants in *MEN1* gene as the cause of MEN1 in 1997 (2). In this report, we compare the clinical characteristics of genotype-negative versus genotype-positive MEN1 and investigate the possibility of mosaicism in *MEN1* as a cause of genotype-negative MEN1. We also evaluate the existence of any genotype-phenotype correlations among patients with genetically confirmed MEN1. We hypothesize that the genotype-negative patients with MEN1 are a genetically heterogenous cohort with a distinct phenotype and attempt to identify clinical characteristics that would best distinguish them from genotype-positive patients with MEN1.

## Results

### Variant analysis

**Figure 1:**
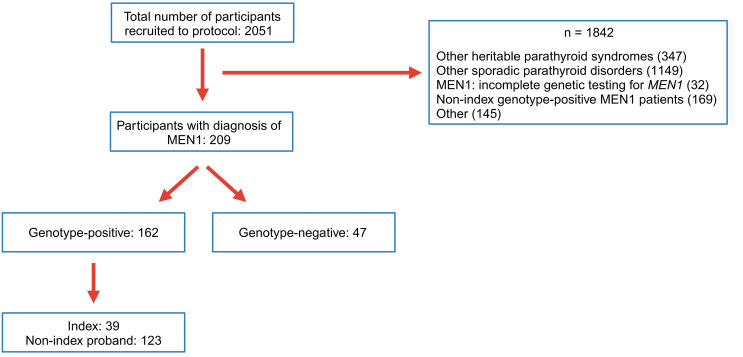
Study schematic. Study schematic for design, sample selection, and study population.

**Figure 2:**
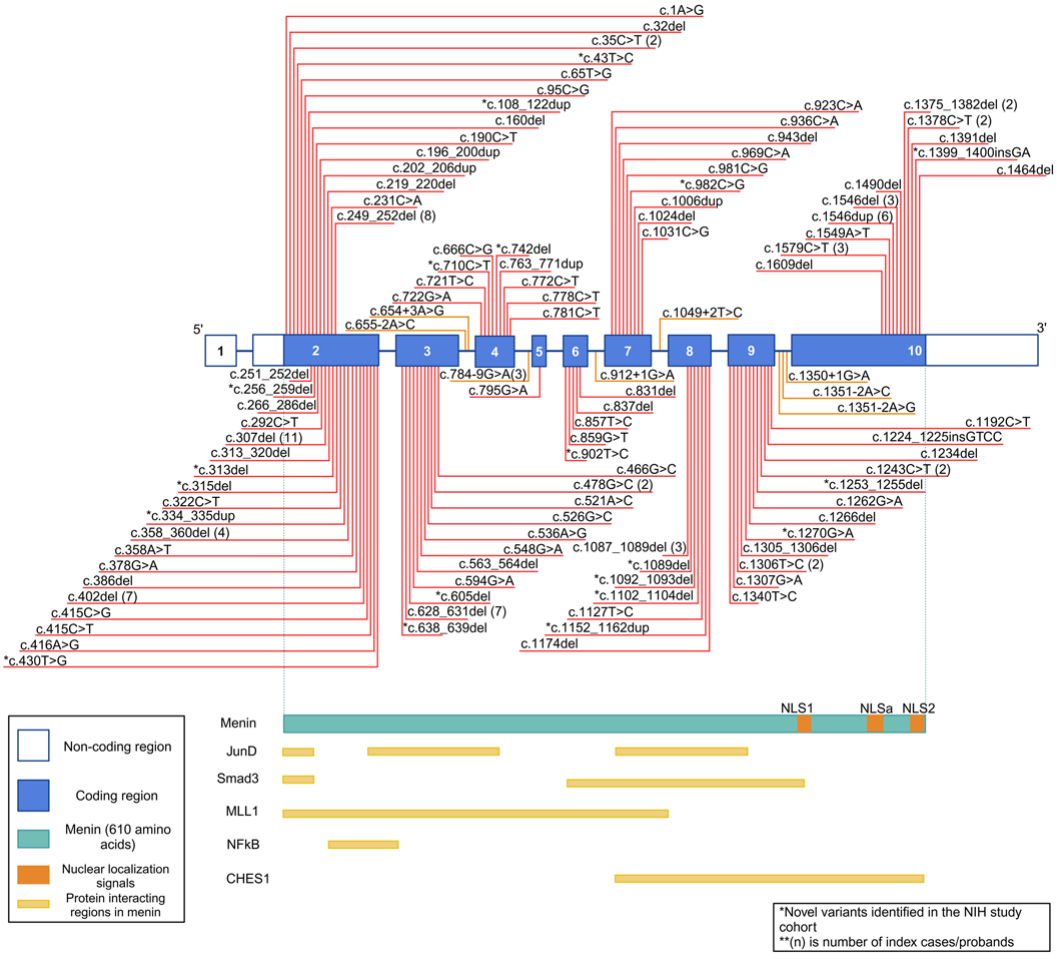
*MEN1* gene schematic with variants noted in our cohort. Figure showing the number of unique variants, number of index patients or probands with the variant, and variant types found on each exon identified in the study. There were 5 whole/partial gene deletions not shown in the figure. The coding region for *MEN1* includes exons 2 through 10 and encodes the protein menin. The gene contains 2 nuclear localization signals (NLSs), NLS1 (amino acids 479–497) and NLS2 (amino acids 588–608), and 1 accessory NLS (aNLS, amino acids 546–572). It interacts with proteins JunD (menin amino acids 1–40, 139–242, and 323–428) (51), checkpoint kinase 1 (CHES1, menin amino acids 428–610) (52), Smad3 (menin amino acids 1–40, 278–477) (53), NF-κB (menin amino acids 276–479) (51), and mixed lineage leukemia protein-1 (MLL1, menin amino acids 1–350) at sites shown in the figure in yellow. Exon 1 is noncoding and includes residues c.–110 to c.–24; exon 2 (c.-23 to 445); exon 3 (c.446 to 654); exon 4 (c.655 to 783); exon 5 (c.784 to 824); exon 6 (c.825 to 912); exon 7 (c.913 to 1049); exon 8 (c.1050 to 1185); exon 9 (c.1186 to 1350); and exon 10 (c.1351 to 1833). Noncoding regions in exon 2 and 10 are indicated. Variant nomenclature is as per *MEN1* sequence encoding the 610–amino acid version of menin, accession number NM_130799.2. See Supplemental Table 2 for consequences of novel variants observed in the cohort. Created with BioRender.com.

We identified 162 index patients or probands (123 probands, 39 index) with genetically confirmed MEN1 (Figure 1) and 47 patients who were genotype negative but met the clinical (*n* = 44/47) or familial (*n* = 3/47) criteria for MEN1 diagnosis (47/209, 22%). Index patient was defined as the first patient in the kindred to develop MEN1-related manifestations. If the index patient in the kindred had not been evaluated at our center, the proband, defined as the first patient from the kindred to be evaluated at our center, was identified and included in this analysis. In addition to *MEN1*, sequencing and deletion-duplication analyses were performed on additional genes per clinical indication: *CDKN1B*: 39/47 (83%), *CDC73*: 29/47 (62%), *CASR*: 27/47 (57%), *RET*: 24/47 (51%), *GCM2*: 18/47 (38%), *AIP*: 14/47 (30%), *VHL*, *NF1*, *TSC1*, *TSC2*, *GNA11*, and *AP2S1*: 13/47 (28%). Patients with positive variants in any of these genes have been excluded from this analysis. There was a predominance of female patients in both genotype-positive (98/162, 60%) and genotype-negative (36/47, 77%) groups (*P* — not significant). The genotype-positive group consisted of 106 unique *MEN1* variants — 78/106 (70%) truncating (40 frameshift insertion or deletion, 25 nonsense, 8 splice site, and 5 whole or partial gene deletions) and 33/106 nontruncating variants (27 missense and 6 in-frame insertions or deletions) (Figure 2). We observed the maximum number of variants in exon 2 of the gene, followed by exons 9, 10, and 3 (Supplemental Table 1; supplemental material available online with this article; https://doi.org/10.1172/jci.insight.176993DS1). However, after adjusting for exon size, variants were noted to be uniformly spread across the entire coding region (Supplemental Figure 1). Some of the variants observed in our cohort have not been previously reported nor their pathogenicity definitively established to our knowledge. We present available in silico, population-, and individual-level data to support the pathogenicity of these potentially novel variants (Supplemental Table 2).

### Genotype-phenotype correlation in genotype-positive and genotype-negative patients

**Figure 3:**
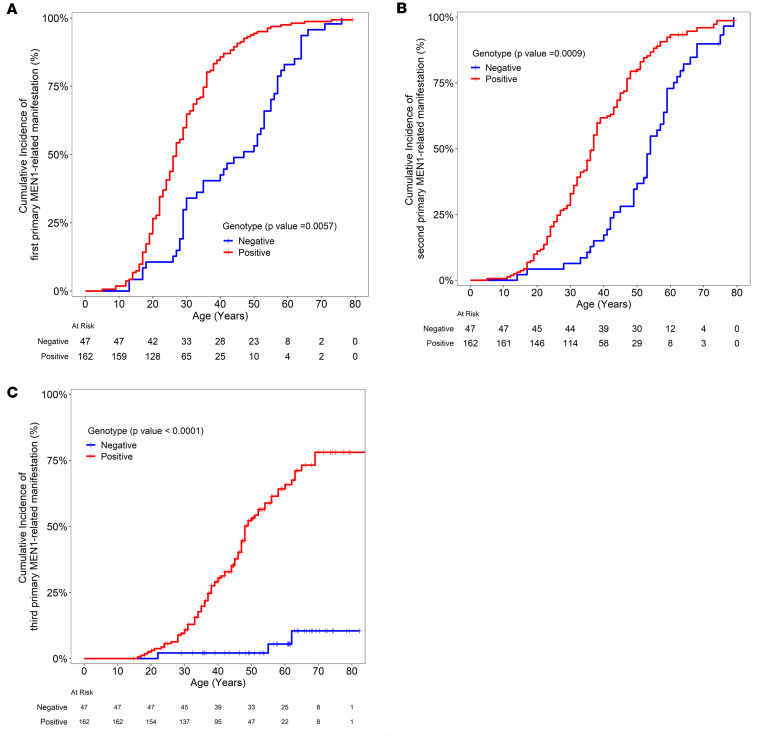
Age-related penetrance of primary endocrine tumors by genotype status. Kaplan-Meier curve relating age at diagnosis on the *x* axis to cumulative occurrence of first (**A**), second (**B**), and third (**C**) primary MEN1-related endocrine tumor (irrespective of the site of the primary tumor) on the *y* axis for both genotype-positive and genotype-negative groups. Each step down on the curve refers to a diagnosis of the respective tumor within the group. Crosses refer to age at last follow-up of a patient with no diagnosis of the relevant tumor in the respective group. The number of individuals in each category at each time point is depicted in the table below the curve. *P* value denotes statistical comparison for significant differences between the groups.

Mean age at last follow-up for patients with genotype-positive disease was 49 (±16) years and for those with genotype-negative disease was 57 (±14) years (*P* = 0.001). Mean duration of follow-up for patients with genotype-positive disease was 10 (±11) years and for those with genotype-negative disease was 5 (±6) years (*P* < 0.0001). Mean age at presentation with first primary manifestation of MEN1 was 27 (±12) years in the genotype-positive group versus 43 (±18) years in the genotype-negative group (*P* < 0.0001). Kaplan-Meier curve analysis showed that patients with genotype-positive disease had a significantly higher age-related penetrance of their first (*P* = 0.004), second (*P* = 0.0009), and third (*P* < 0.0001) primary endocrine tumors than those with genotype-negative disease (Figure 3, A–C).

#### Genotype-phenotype correlation: pituitary.

**Figure 4:**
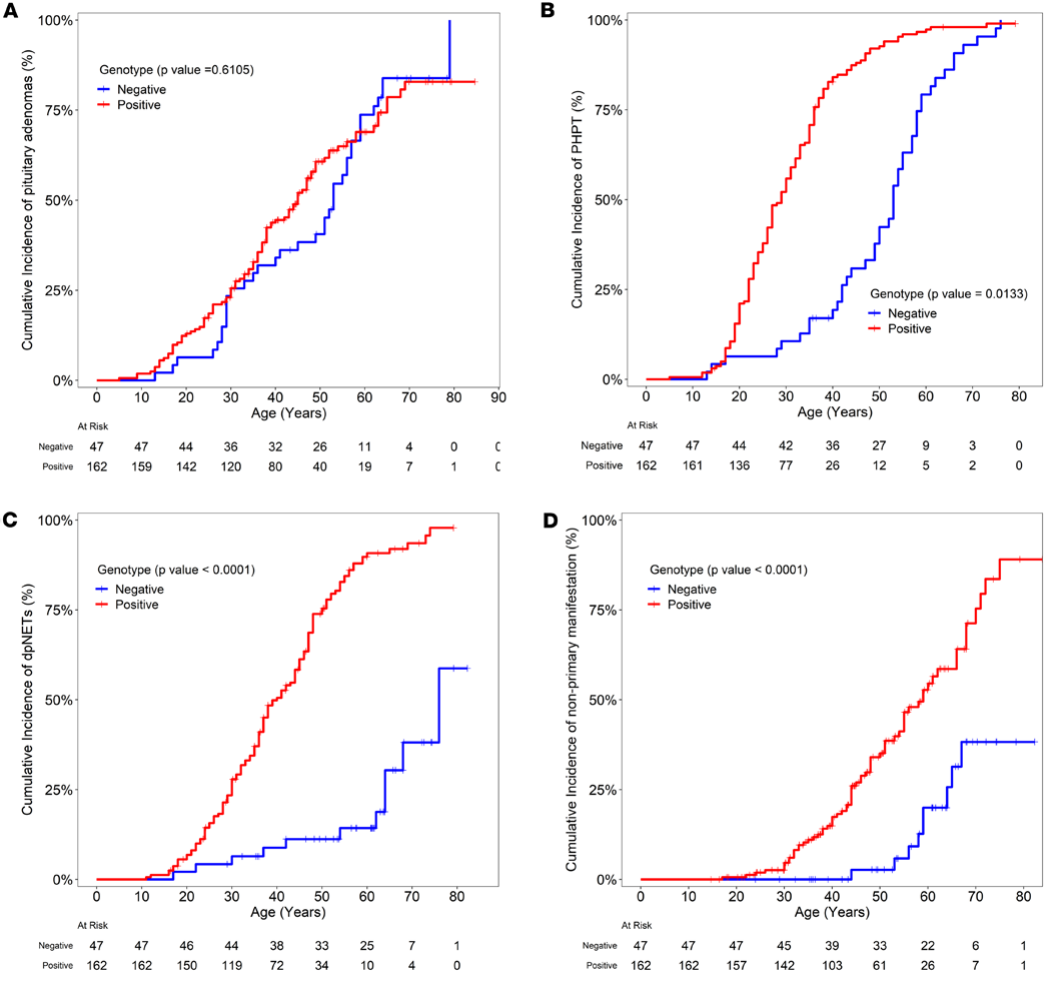
Age-related penetrance of individual primary and nonprimary manifestations by genotype-status. Kaplan-Meier curve relating age at diagnosis on the *x* axis to cumulative occurrence of primary pituitary adenomas (**A**), hyperparathyroidism (**B**), dpNETs (**C**), and nonprimary manifestations such as lung NET or thymic or adrenal tumors (**D**) on the *y* axis for both genotype-positive and genotype-negative group of patients. Each step down on the curve refers to a diagnosis of the respective tumor within the cohort. Crosses refer to age at last follow-up of a patient with no diagnosis of the relevant tumor in the respective curve. The number of individuals in each category at each time point is depicted in the table below the curve. *P* value denotes statistical comparison for significant differences between genotype-positive and genotype-negative groups.

Among patients with genotype-positive disease, 103/162 (64%) were diagnosed with a pituitary adenoma versus 38/47 (81%) patients in the genotype-negative group (*P* — not significant). Pituitary imaging was available in 135/162 (83%) genotype-positive and 37/47 (79%) genotype-negative patients (MRI pituitary/CT sella in case of metal-containing implants). Mean age of diagnosis with pituitary adenoma was 35 (±15) years for genotype-positive and 45 (±16) years for patients with genotype-negative disease (*P* = 0.001; Table 1, Supplemental Figure 2). Kaplan-Meier curve analysis showed no difference in the age-related penetrance of pituitary adenoma in genotype-positive versus genotype-negative disease (Figure 4A). Of those diagnosed with pituitary adenoma with available data about size of the adenoma, 40/97 (41%) in the genotype-positive group and 16/32 (50%) in the genotype-negative group developed a macroadenoma (tumor > 1 cm; *P* — not significant). Similarly, of those diagnosed with pituitary adenoma with available data about tumor function, 52/102 (49%) in the genotype-positive group and 25/38 (66%) in the genotype-negative group developed a functioning pituitary adenoma (*P* — not significant). There was a higher frequency of acromegaly among patients with genotype-negative disease (8/47, 17%) as compared with those with genotype-positive disease (1/162, <1%, *P* < 0.0001). Of the 8 patients with acromegaly in the genotype-negative group, 4 had been tested for germline variants in *AIP* (sequencing and deletion-duplication analysis) and tested negative. Among patients with genotype-positive disease, there was no association between exon location of the variant, variant type (truncating vs. nontruncating), or variants affecting the central cavity of menin and diagnosis of pituitary adenoma, age at diagnosis, or size or functional status of the pituitary adenoma (Supplemental Tables 3–5).

#### Genotype-phenotype correlation: parathyroid.

There was a strong preponderance of PHPT in both genotype-positive and genotype-negative groups, 157/162 (97%) patients in genotype-positive versus 44/47 (94%) in genotype-negative groups (*P* — not significant). However, 128/157 (81%) genotype-positive patients who developed PHPT had recurrent hyperparathyroidism in comparison with only 3/44 (7%) patients in the genotype-negative group (*P* < 0.0001). Mean age at diagnosis with PHPT was 30 (±11) years in patients with genotype-positive versus 50 (±15) years in those with genotype-negative disease (*P* < 0.0001; Table 1 and Supplemental Figure 2). Kaplan-Meier curve analysis showed that patients with genotype-positive disease had a significantly higher age-related penetrance of PHPT than those with genotype-negative disease (*P* = 0.0133, Figure 4B). Among patients with genotype-positive disease, there was no association between the presence of an underlying truncating germline *MEN1* variant, variants affecting the central cavity of menin, or exon location of the variant and diagnosis of PHPT, age at diagnosis, or recurrence (Supplemental Tables 3–5).

#### Genotype-phenotype correlation: dpNETs.

Through the duration of their follow-up, 134/162 (82%) patients in the genotype-positive group and 11/47 (23%) patients in the genotype-negative group developed dpNETs (*P* < 0.0001) (Figure 4C). Abdominal imaging was available in 138/162 (85%) genotype-positive and 42/47 (90%) genotype-negative patients (MRI/CT abdomen or somatostatin receptor–based imaging). However, history and a fasting serum gastrin and glucose were available for all patients. Pancreatic NETs (PNETs) were seen in 117/138 (85%) patients with genotype-positive disease versus 10/42 (24%) patients in the genotype-negative group with available abdominal imaging (*P* < 0.0001). A total of 24/162 (15%) patients in the genotype-positive group versus 3/47 (6%) patients in the genotype-negative group developed insulinomas (*P* — not significant). Similarly, 77/162 (48%) patients in the genotype-positive group versus 2/47 (4%) patients in the genotype-negative group were diagnosed with Zollinger-Ellison syndrome (ZES or gastrinomas; *P* < 0.0001), typically seen in the duodenum. There were 36/134 (27%) patients in the genotype-positive group versus 2/11 (18%) in the genotype-negative group who developed distant metastasis (*P* = not significant). Mean age of diagnosis with dpNET was 38 (±13) years for patients with genotype-positive disease versus 49 (±20) years in patients with genotype-negative disease (*P* — not significant; Table 1 and Supplemental Figure 2). Kaplan-Meier curve analysis showed that patients with genotype-positive disease had significantly higher age-related penetrance of dpNETs in comparison with those with genotype-negative disease (*P* < 0.0001, Figure 4C). Among patients with genotype-positive disease, we noted a difference in the association between exonic location of the variants and the frequency of distant metastases. On further interrogating this observation for direction, we found a decreased frequency of distant metastases among patients with germline *MEN1* variants in exons 2 (13/49 patients, *P* < 0.001) and 3 (1/18 patients, *P* < 0.001). There was no difference in the frequency of distant metastases between truncating versus nontruncating variants in these exons. Variants in exons 2 and 3 accounted for 49% of patients in our cohort. An association with increased risk of distant metastases was not observed with any of the exons. There was no association between presence of an underlying truncating germline *MEN1* variant or variants affecting the central cavity of menin; diagnosis of dpNET, insulinomas, or ZES; and age of dpNET diagnosis (Supplemental Tables 3–5).

#### Genotype-phenotype correlation: thymic and lung NETs.

Chest imaging was available for 116/147 (79%) genotype-positive and 22/47 (47%) genotype-negative patients (MRI or CT of the chest or somatostatin receptor–based chest imaging), indicating markedly fewer chest imaging studies obtained in patients with genotype-negative disease. Of the available data, lung and thymic NETs were observed only in genotype-positive patients, specifically 29/162 (18%) with lung NETs and 5/162 (3%) with thymic tumors (4/5 with thymic NETs and 1 with thymoma; Table 1 and Supplemental Figure 2). All 4 patients with thymic NETs had underlying truncating germline *MEN1* variant while the patient with thymoma had a nontruncating variant. Mean age at diagnosis was 51 (±11) years for lung NETs and 48 (±14) years for thymic NETs. Kaplan-Meier curve analysis showed that patients with genotype-positive disease had significantly higher age-related penetrance of nonprimary manifestation consisting of lung, thymic, and adrenal NETs in comparison with those with genotype-negative disease (Figure 4D). There was no association between the presence of an underlying truncating germline *MEN1* variant, variants affecting the central cavity of menin, or exon location of the variant and diagnosis or age at diagnosis of these foregut NETs (Supplemental Tables 3–5).

#### Genotype-phenotype correlation: adrenal.

Adrenal lesions were noted in 52/162 (32%) patients in the genotype-positive group and 9/47 (19%) patients in the genotype-negative group (*P* — not significant). Mean age at diagnosis of adrenal lesions in genotype-positive patients was 45 (±13) years and 58 (±7) years in the genotype-negative group (*P* = 0.0001; Table 1 and Supplemental Figure 2). Of those with available data about the functional status of the tumor, 5/46 (11%) patients had functional adenomas in the genotype-positive group (3 cortisol-producing, 2 pheochromocytomas) (20) versus 1/7 (14%, aldosterone-producing) in the genotype-negative group (*P* — not significant). There was no association between the presence of an underlying truncating germline *MEN1* variant, variants affecting the central cavity of menin, or exon location of the variant and diagnosis, age at diagnosis, or functional status of these adrenal lesions in patients with genotype-positive disease (Supplemental Tables 3–5).

#### Genotype-phenotype correlation: skin.

Angiofibromas were seen in 55/162 (34%) patients in genotype-positive disease and 1/47 (2%) with genotype-negative MEN1 (*P* < 0.0001). Similarly, collagenomas were seen in 35/162 (22%) genotype-positive patients and 2/47 (4%) with genotype-negative disease (*P* = 0.0051). However, the prevalence of lipomas was not significantly different between the 2 groups (46/159 genotype-positive patients and 8/47 with genotype-negative disease, *P* — not significant). Together, angiofibromas or collagenomas were described in 66/162 (41%) patients with genotype-positive disease and 3/47 (6%) patients with genotype-negative disease (*P* < 0.0001; Table 1 and Supplemental Figure 2). Among the genotype-positive patients, none of the skin lesions showed an association with an underlying truncating versus nontruncating germline *MEN1* variant or exon location of the variant (Supplemental Tables 3–5). However, patients with germline variants affecting the central cavity of menin appeared to be at lower risk of developing angiofibromas (28% versus 45%, *P* = 0.02) and collagenomas (15% versus 34%, *P* = 0.005, Supplemental Tables 3–5).

#### Genotype-phenotype correlation: other rare tumors.

Meningioma was diagnosed in 2% of patients in both genotype-positive and genotype-negative groups (3/162 vs. 1/47). Spinal ependymoma was diagnosed in 1/162 (<1%) patient with genotype-positive disease and none with genotype-negative disease. Leiomyomas were noted in 32/162 (20%) patients with genotype-positive disease versus 7/47 (15%) patients with genotype-negative disease (*P* — not significant). Of the 32 patients with leiomyomas in the genotype-positive group, 30/32 (94%) had uterine leimyomas, 2 had esophageal leimyomas, while 1 had both uterine and esophageal leimyomas. A total of 24/32 (75%) patients with genotype-positive MEN1 needed resection of their leiomyomas while the remaining patients were conservatively managed through their last follow-up. All 7 patients in the genotype-negative group with leiomyomas developed uterine leiomyomas. Of these, 6/7 (86%) had undergone resection at last follow-up.

### Comparing by presenting manifestation: parathyroid and pituitary tumor versus dpNET groups

To characterize our cohort by initial presenting manifestation, we compared patients who presented with parathyroid and pituitary tumors versus those presenting with dpNETs in combination with parathyroid or pituitary tumors (Table 2 and Supplemental Figure 3). There were 53/162 (33%) patients who presented with dpNETs versus 11/47 (23%) in the genotype-negative group (*P* — not significant). A significant proportion of the genotype-negative group consisted of patients presenting with parathyroid and pituitary tumors (36/47, 77% vs. 11/47, 23% presenting with dpNETs). None of the genotype-negative patients who presented with parathyroid and pituitary tumors developed dpNETs. In contrast, 81/109 (74%) patients with genotype-positive disease presenting with parathyroid and pituitary tumors developed dpNETs. Similarly, there were 3/11 (27%) patients in the genotype-negative group versus 31/53 (58%) in the genotype-positive group presenting with dpNETs who developed a third primary MEN1-related endocrine tumor.

### Receiver operating characteristic curve analysis

Based on background knowledge and the above analysis, age at presentation with first MEN1-related primary manifestation, recurrent PHPT, diagnosis of dpNET, and diagnosis of collagenoma or angiofibroma were included as independent variables for regression modeling. Multivariable binomial logistic regression analysis showed that age ≤ 39 years at presentation (OR = 8, 95%, CI: 2–28, *P* = 0.0009), recurrent PHPT (OR = 67, 95% CI: 14–335, *P* < 0.0001), diagnosis of dpNET (OR = 18, 95% CI: 5–64, *P* < 0.0001), and diagnosis of collagenoma or angiofibroma (OR = 6, 95% CI: 1–32, *P* = 0.055) were predictive of genotype-positive disease. Receiver operating characteristic (ROC) analysis of age at presentation and genotype status showed good accuracy (ROC area under the curve [AUC] 0.75, 95% CI: 0.67–0.84, Supplemental Figure 4), supporting that age at presentation provides an informative model for differentiating genotype-positive from genotype-negative disease. Based on this analysis, age ≤ 39 (sensitivity 86%, specificity 60%) provided the best discrimination between genotype-positive versus genotype-negative disease. With every 1-year increase in age of presentation with first MEN1-related primary manifestation, the odds of genotype-positive disease decreased by 7% (CI 5%–10%). Age ≤ 39 provided a positive predictive value (PPV) of 88% and a negative predictive value (NPV) of 54% for predicting the risk of genotype-positive disease.

### Development of a weighted scoring system to predict genotype-positive MEN1

The regression β-coefficient values of the predictor variables in the multiple logistic regression model were rounded off to the nearest integer (age ≤ 39 years at presentation with first MEN1-related primary manifestation = 1.05, recurrent PHPT = 2.10, diagnosis of dpNET = 1.44, and diagnosis of collagenoma or angiofibroma = 0.86). The scores of each item in the scoring system were defined as 2 points for recurrent or multigland parathyroid disease, 1 for presence of dpNET, 1 for age at presentation with first MEN1-related primary manifestation ≤ 39, and 1 for presence of angiofibroma or collagenoma (Table 3). This scoring system allowed a total score of 5 points. According to ROC curve, the cutoff value of 2 points yielded the largest area under ROC 0.95 (95% CI: 0.92–0.98) with a sensitivity of 95% and specificity of 83%. A score of ≥2 provided PPV of 95% and NPV of 83%. The mean values with 95% CI using the bootstrap samples were obtained. The mean sensitivity was 86.6% (95% CI: 75.9–97.5), the mean specificity was 90% (95% CI: 79.8–97.9), the mean PPV was 97% (95% CI: 93.3–99.3), and the mean NPV was 69% (95% CI: 50.7–91.5). Therefore, total score of 2 was selected as the optimal cutoff value of the scoring system (Supplemental Figure 5). Of note, these scoring criteria to predict risk of genotype-positive MEN1 were developed for application in patients who present with 2 or more primary MEN1-related endocrine tumors or present with 1 primary MEN1-related endocrine tumor without any family history of genetically confirmed MEN1 in a first-degree relative.

### Somatic or germline mosaicism in genotype-negative patients

Seventeen of 47 patients with genotype-negative disease and available tumor(s) from 1 or more of the 3 primary MEN1-related manifestations were identified. Of these, 9/17 had tissue available from more than 1 primary tumor while 8/17 had tissue from a single primary tumor. Whole-exome sequencing (WES) analysis of 28 (2 additional tumors underwent multiplex PCR testing for *MEN1*) tumors from 17 patients did not reveal any recurrent variants in 2 or more tumors or tumor and germline DNA. Among the candidate genes analyzed, heterozygous variants in *MEN1*, *CDC73*, or *CDKI* were observed in different tumors, and 2 homozygous *MEN1* variants were observed in patient DK-2127 (Supplemental Table 6). However, there was no indication of mosaicism for these variants explaining the disease-related manifestation because none were seen in second tumor(s) from the same patient or in the corresponding germline DNA (Supplemental Figure 6).

## Discussion

Our findings from a well-characterized cohort of 162 index patients with genotype-positive MEN1 and 47 patients with genotype-negative disease verify that patients with genotype-positive disease have a distinct clinical course with multigland or recurrent parathyroid tumors, higher frequency of dpNETs, younger age at initial presentation with MEN1-related manifestation, and the presence of angiofibromas or collagenomas. We propose a weighted scoring system to refine the current diagnostic criteria to establish a diagnosis of genotype-positive MEN1 based on clinical characteristics.

Our findings demonstrating a distinct clinical course for patients with genotype-negative MEN1 are consistent with published findings from 2 large independent cohorts of patients with MEN1 (11, 17). The striking similarities in the generated Kaplan-Meier curves from all 3 of these large independent cohorts underscore the robustness of these findings. The consistency across these 3 large independent cohorts strengthens the argument that genotype-negative MEN1 represents a distinct disease, raising uncertainty about the benefits of lifetime surveillance in patients with genotype-negative MEN1.

MEN1 has been conventionally characterized as a heritable endocrine neoplasia, which may be familial or arise de novo. A diagnostic genetic test positive for a germline *MEN1* variant is the gold standard for diagnosis of MEN1 and the only available confirmatory test. The finding of a germline *MEN1* variant establishes the diagnosis, reinforcing the need for a surveillance plan for tumor detection, while a negative test relieves the individuals and their children of the emotional burden of diagnosis and reduces the cost of unnecessary biochemical and radiological testing (12, 21). When genetic testing is not available, a diagnosis of MEN1 is made by the presence of at least 2 primary MEN1-related manifestations or 1 primary manifestation in case of positive family history in a first-degree relative. However, this criterion has low specificity for identification of genotype-positive disease (11, 17). About 26% of patients with 2 MEN1-related primary manifestations have genotype-positive disease among those without any family history of MEN1 (22). We propose a predictive weighted risk score to further refine the clinical criterion by inclusion of additional characteristics: age of first primary MEN1-related manifestation ≤ 39, presence of recurrent PHPT or multigland parathyroid disease, presence of dpNETs, and presence of angiofibromas or collagenomas. Since recurrence is a factor of the number of glands resected during parathyroidectomy, we incorporated multigland disease (adenoma/hyperplasia noted in multiple glands on surgical pathology) as an equivalent of recurrent PHPT, the strongest predictor of genotype-positive disease as evidenced by our findings and published literature (23). There were 3 patients with genotype-negative disease who met the current diagnostic criteria based on the familial criteria for diagnosis of MEN1 (1 or more primary MEN1-related endocrine tumors in the presence of a positive family history of MEN1 in a first-degree relative, irrespective of genetic confirmation). Neither of the 3 patients had a family history of genetically confirmed MEN1. Given that genetic testing is the only confirmatory test to diagnose MEN1, we propose further refinement of these criteria as 1 MEN1-related primary manifestation in a patient with a family history of genetically confirmed MEN1 in a first-degree relative. Our findings indicate a prevalence of genotype-negative MEN1 of 22% among all unrelated patients with MEN1, consistent with published literature (1).

Several speculations have been proposed to explain the lack of a germline *MEN1* variant in patients with genotype-negative disease. First, these patients may carry a variant in regions of *MEN1* that is not covered by current genetic testing methods like untranslated, intronic, and regulatory regions. However, targeted next-generation sequencing of the entire 7.2-kilobase genomic region of *MEN1* revealed no point or short indel mutations in noncoding regions of *MEN1* in 76 patients with the disease (49 familial, 27 sporadic), indicating that such variants may be rare or nonexistent (24). Alternatively, these patients may carry a germline variant in an alternative gene in the same pathway as *MEN1*, resulting in the same pathological phenotype. However, efforts to identify such a gene(s) have remained unsuccessful (25). Rarely, patients may test positive for variants in genes that cause manifestations similar to MEN1, for example, *CDKN1B* or other *CDKI* genes (18, 26), *CDC73* (19), *CASR* (25), *GNA11*, *AP2S1* (27), *GCM2*, *AIP* (18, 28), *VHL*, *TSC1*, *TSC2*, or *NF1*. Third, tumors in patients with genotype-negative MEN1 may result from mosaic *MEN1* variants or other novel genes (29). The only study that investigated tumors from patients with genotype-negative MEN1 included samples from only 6 patients with a single tumor from each, limiting its broad extrapolation (25). The study authors concluded that recurrent variants in *MEN1* or another novel tumor suppressor gene explaining the MEN1 phenotype in genotype-negative patients was likely to be rare. Nevertheless, epigenetic changes that can affect gene expression have not been investigated in genotype-negative MEN1. Pragmatically, it is possible that the occurrence of 2 MEN1-associated primary tumors in genotype-negative patients is a true sporadic co-occurrence with no underlying germline predisposition but rather independent somatic events.

Our evaluation of 30 tumors from 17 patients with genotype-negative MEN1 did not provide evidence for mosaicism in *MEN1* or other candidate genes. Recent case reports describe mosaicism for *MEN1* (both truncating and nontruncating variants) at an allele frequency as low as 2.5% (30, 31). Based on available information, the disease course in patients with mosaic *MEN1* variants appears to mimic that of patients with genotype-positive disease with no reported differences in age of onset or frequency of dpNETs (32, 33). Furthermore, the severity of MEN1 does not appear to correlate with allele frequency as thymic NETs and dpNETs have been reported in patients with variant allele frequency less than 10% (30). Although these cases appear to be rare, detection of such cases can be a balancing act between lowering the threshold for variant detection without introducing sequencing artifacts. The clinical context, such as family history, or high predictive risk score and demonstration of the variant with biallelic *MEN1* loss in the patient’s tumor(s) can help confirm the diagnosis. Biallelic *MEN1* loss was noted in a parathyroid tumor from patient DK-2127, but neither of the 2 variants were noted in the patient’s germline DNA on WES or Sanger sequencing or in the germline DNA of the patient’s sister with genotype-negative MEN1. Our cohort comprised 39/162 (24%) index patients, which suggests a de novo origin of *MEN1* variant based on available family history (genetic testing on parents was not available). This contrasts with the reported 10% prevalence of de novo disease and may represent referral bias to our institute (12). Parental mosaicism has been reported in a patient with apparent de novo MEN1 (26). Patients with *MEN1* mosaicism may transmit the variant to offspring in the form of a constitutional variant depending on the proportion of germ cell progenitors that harbor the variant. Our findings indicate that *MEN1* mosaicism is rare and likely constitutes a minor proportion of genotype-negative disease. Thus, genotype-negative MEN1 likely represents a heterogenous group of patients with a clinical course distinct from those with genotype-positive disease.

Few studies have described a genotype-phenotype correlation in patients with genotype-positive MEN1 (12, 21, 34–37). We observed a reduced risk of distant metastases in patients with germline variants in exons 2 and 3. This contrasts with reports of increased risk of distant metastases with variants in exon 2 reported in some prior publications (34, 35, 38, 39). Germline variants in *MEN1* exon 2 have been reported to be the most frequent underlying cause of MEN1 (40) with possibly an increased risk of dpNET formation (41). Given conflicting observations about genotype-phenotype correlation in MEN1 in published literature, large collaborative studies focusing on MEN1 patients with distant metastases allowing for a greater sample size of such patients and uniform surveillance testing are needed to firm up such observations. In general, mutant protein resulting from missense and in-frame insertion or deletion has approximately the same molecular weight as the wild-type protein, in contrast with truncating variants, which may result in no protein synthesis or the expression of a lower molecular weight protein. It seems intuitive that patients with missense variants would have a milder phenotype. However, our findings from a well-characterized cohort of 162 index patients and probands does not support this hypothesis with 30/121 (25%) patients with truncating variants versus 6/41 (15%) patients with nontruncating variants developing distant metastasis from a MEN1-related NET (*P* — not significant). We also looked to see if variants affecting the central cavity of the menin 3D structure that facilitates protein-protein interaction showed any association with clinical features. To our surprise, we noted a decreased frequency of angiofibromas and collagenomas in patients with variants affecting central cavity of the menin structure, which could suggest an alternative pathomechanism for MEN1-related skin manifestations. However, not all our patients underwent a systematic dermatologic exam, and hence, these findings should be interpreted with caution.

We observed an association between acromegaly and genotype-negative MEN1, as seen in several prior studies (17, 26, 28, 42). However, given the overall rarity of MEN1-related acromegaly, this variable was not included in regression modeling. Similarly, thymic tumors were seen only in patients with genotype-positive disease but were not included in regression analysis given their overall rarity. Lung NETs were only observed in the genotype-positive group in our cohort, consistent with reports from other centers (11, 17). However, given its relatively low preponderance in comparison with neuroendocrine carcinoma and potential overlap in imaging characteristics between the two, it was not included as a predictor for regression analysis. Lung NETs comprise 2% of lung neuroendocrine neoplasms, of which 5% of patients have MEN1 (43).

Although diagnosis of angiofibroma or collagenoma attained only borderline statistical significance as a predictor, the actual frequency of these cutaneous lesions in our cohort is likely to be higher as not all patients underwent a comprehensive skin examination. Previous reports from our center, which included comprehensive dermatologic evaluation of patients with MEN1, had revealed a prevalence of 64%–88% for angiofibromas and 62%–72% for collagenomas (44, 45). Both lesions can occur sporadically or in the context of other heritable syndromes, such as tuberous sclerosis complex. The penetrance of these lesions increases with age in MEN1 (85% for angiofibromas and 70% for collagenomas by age 40). Early detection of these lesions may have a role in the diagnosis of MEN1 in the context of a MEN1-related primary manifestation (sensitivity 75%, specificity 95% in the presence of gastrinomas) (45).

Among the patients with genotype-negative disease, there were only 3/47 patients (all 3 presented with dpNETs) who developed a third primary MEN1-related endocrine tumor in contrast with 88/162 (54%) patients in the genotype-positive group. Our findings of no dpNETs in genotype-negative patients presenting with parathyroid and pituitary tumors is consistent with prior reports (11, 17). Overall, it appears that the risk of developing a third primary endocrine tumor in patients with genotype-negative disease is low. Furthermore, the value in diagnosing a nonfunctional pituitary microadenoma, PHPT in the absence of target organ effects, or a low-grade, small, nonfunctional PNET, particularly at an older age, remains uncertain. Thus, it seems increasingly apparent that patients with genotype-negative MEN1 are unlikely to derive a significant benefit from lifetime surveillance testing for MEN1-related manifestations.

Our study represents genotype-phenotype correlation analysis from a large, well-characterized cohort of patients with MEN1. The inclusion of only index patients or probands with genotype-positive disease mitigates the influence of intrafamilial exposures and detection bias from surveillance, particularly for analysis of age at diagnosis. Furthermore, sequencing and deletion-duplication analysis were performed not only for *MEN1* but also for additional genes known to frequently present with MEN1-like manifestations (*CDKN1B*, *CDC73*, *CASR*, *RET*, *GCM2*, *GNA11*, *AIP*, *VHL*, *NF1*, *TSC1*, *TSC2*, and *AP2S1*) in most patients. Nevertheless, our study has some limitations. First, this is a retrospective study with cases limited to a single institution. Further experience in other institutions will be required to confirm the usefulness of the scoring system. Second, our data regarding phenotype are limited by follow-up duration and available documentation. Nevertheless, the mean age at last follow-up for patients with genotype-negative disease was 57 (±14) years [and 49 (±16) years for genotype-positive disease], which is greater than the mean age of presentation with most MEN1-related manifestations. We were unable to assess mortality in our cohort because of lack of such data. Furthermore, given the duration of the study and its retrospective nature, it is likely that the evaluation for MEN1-related tumors was not performed systematically and similarly for both groups of patients, particularly for nonconventional tumors such as skin, thymus, and lung. Third, as a study spanning several decades, improvements in assays and imaging techniques can confound disease detection. Moreover, advances in knowledge about the disease can influence physicians’ threshold to obtain further laboratory testing or scans. Fourth, the weighted risk score/prediction model for genotype-positive MEN1 will need to be validated in other independent cohorts. However, with increasing access to genetic testing, such a predictive score model is expected to have limited utility. Last, any differential effect of other systemic influences, for example, environmental toxicants, medications, etc., on the 2 groups could not be accounted for.

We conclude that patients with genotype-negative MEN1 have a distinct clinical course in comparison with genotype-positive disease. Whether genotype-negative disease should even be described as “MEN1” in the context of its conventional description and increasing accessibility to genetic testing remains questionable. *MEN1* mosaicism is rare in patients with genotype-negative disease. Genetic testing is the only confirmatory test for diagnosis of MEN1. The incorporation of a weighted predictive scoring system to diagnose genotype-positive MEN1 among patients with at least 2 primary MEN1-related manifestations assuming negative family history can increase the predictability of genotype-positive disease. Patients with germline *MEN1* variants in exons 2 and 3 may have a decreased predisposition to distant metastases. Further research to develop predictors of aggressive genotype-positive MEN1 is warranted. An improved understanding of the pathophysiology and natural history of genotype-negative MEN1 is needed so that appropriate surveillance recommendations can be established for this group of patients.

## Methods

### Sex as a biological variable.

Our study examined both male and female patients. Sex was not considered as a biological variable for these analyses.

### Patient selection.

We identified patients with a diagnosis of MEN1 seen under the Natural History Study of Parathyroid Disorders protocol (ClinicalTrials.gov Identifier: NCT04969926) between 1977 and 2022 and subsequently extracted data pertaining to their genetic testing and clinical course. MEN1 was diagnosed in all patients who met the clinical (at least 2 primary MEN1-related tumors), familial (at least 1 primary MEN1-related tumor in the context of a first-degree relative with a clinical diagnosis of MEN1), or genetic criteria (demonstration of a pathogenic variant in *MEN1*) (12). Genetic testing was performed through a Clinical Laboratory Improvement Amendments–certified commercial laboratory in conjunction with testing performed in our research laboratory in the years before testing became commercially available. Negative genetic testing was defined as lack of mutation detection in *MEN1* on both sequencing and deletion/duplication analyses. Frameshift insertion or deletion, nonsense mutations, whole or partial gene deletion, and splice site mutations were categorized as truncating while missense and in-frame insertion or deletion were categorized as nontruncating variants. Variants were categorized as novel in the absence of their annotation in VarSome and ClinVar in conjunction with published literature (46). Structural effects of missense variants were analyzed using HOPE (47).

### Phenotyping.

Patients suspected to have or diagnosed with MEN1 underwent comprehensive phenotyping, including laboratory tests (routine blood tests including markers of calcium homeostasis, pituitary function tests, and fasting serum gastrin) and imaging of pituitary, chest, and abdomen. Patients with a history of ZES or those with symptoms suggestive of ZES were evaluated by a gastroenterologist and further workup, with secretin stimulation test, basal gastric acid output, or esophagogastroduodenoscopy with biopsy pursued as clinically appropriate. Duodenal NETs were considered absent if there were no concerning symptoms of heartburn or acid reflux or serum gastrin levels were normal. Pituitary adenoma was considered negative in the absence of MRI pituitary if there were no concerning symptoms or pituitary function tests were normal. Similarly, lung NETs were considered negative in the absence of any available imaging. For the purposes of these analyses, adrenal abnormality was defined as the presence of discrete adrenal lesions and not just limb thickening. Functional assessment of adrenal nodules was performed as per standard clinical practice. When not performed, the nodule was considered nonfunctional because of lack of suggestive signs and symptoms indicating a low pretest probability. Angiofibromas and collagenomas were considered positive when documented in physical exam for at least 1 visit. When no such documentation was retrieved, these lesions were considered negative. Patient follow-up was individualized based on disease course and access to care.

### Tumor DNA extraction and sequencing.

Genotype-negative patients with available tumor from 1 or more of the 3 primary MEN1 manifestations were identified. Parathyroid tumors from 2 patients with genotype-negative MEN1, which had undergone multiplex PCR sequencing at National Cancer Institute (NCI) Frederick for *MEN1*, were included in this analysis (DK-1301 and DK-1093) (48). One patient (DK-2127) underwent paired tumor germline DNA next-generation sequencing at NCI Laboratory of Pathology according to methods previously described (48). For WES (*n* = 14 patients), tumor DNA isolation from 5 μm FFPE tissue sections was performed with the DNAstorm FFPE DNA extraction kit (Cell Data Sciences). DNA quality control, library preparation, WES, and analysis were outsourced to QuickBiology, Inc. Library sequencing was performed with the Illumina HiSeq Pe150 Platform for 50× mean coverage. The sequence data were aligned to the GRCh38 human reference genome using BWA v0.7.7-r411. PCR duplicates were marked using MarkDuplicates program in Picard-tools-1.115 tool set. GATK v3.2-2 was used for insertion and deletion realignment and base quality recalibration. Pisces was used to call the somatic SNV/indel for single samples. All variants were annotated using the Annovar program. For the analysis of candidate genes, the raw variants were filtered using the following criteria: depth > 15× and tumor allele frequency > 0.05.

### Statistics.

Study data on genotype-negative and index or proband genotype-positive patients was collected on retrospective chart review using Research Electronic Data Capture electronic data capture tools hosted at the NIDDK (49, 50). Continuous outcomes were summarized using descriptive statistics such as mean and SD or median and interquartile range appropriately while categorical outcomes were summarized using frequency and percentages. For continuous outcomes, differences in phenotype between genotype-positive and genotype-negative groups was tested using 2-tailed Student’s *t* test with Satterthwaite approximation if needed or 1-way ANOVA. For categorical variables, χ^2^ test or Fisher’s exact test was used to test for association with genotype. Age-related penetrance of primary and other MEN1-related tumors was compared using the Kaplan-Meier estimator. Comparison of Kaplan-Meier curves was made with log-rank test.

Genotype-phenotype correlation was assessed among index patients with genetically confirmed MEN1 to avoid confounding because of genetic variability from family members and surveillance-related findings, which may be clinically irrelevant. This was investigated using χ^2^ or Fisher’s exact test in 3 ways: (a) association between truncating versus nontruncating variants in *MEN1* gene and the clinical characteristics, (b) association between exon location of the variants and clinical characteristics, and (c) association between variants affecting the central cavity in the menin 3D structure as the protein-interacting region and clinical characteristics. Further, exact binomial test was used for proportion of patients with distant metastases as needed. Both MLL and JunD bind to this pocket in menin (the central cavity formed by the thumb and palm domains, amino acids 102–230 and 231–386, respectively). This pocket facilitates protein-protein interaction with menin.

For the weighted scoring system/prediction model, factors were selected by using simple logistic regression. Preliminary multivariable logistic regression was conducted using selected factors by using simple logistic regression to identify an adjusted association between genotype status and the selected factor. A final multivariable logistic regression was generated after removing any variables that did not demonstrate statistical significance (*P* ≥ 0.05) in the preliminary multivariate logistic regression model. ROC analyses were performed to obtain AUC and its 95% CI. Youden’s J statistic was used for finding the optimal value of the continuous independent variables by finding the cutoff value of the variable having the highest value of J for maximizing distance between true positive and false positive of genotype status. Covariates significantly associated with genotype status in the multivariable logistic regression model were incorporated in the weighted risk score to predict genotype status. Continuous variables were dichotomized using the optimal cut points from the ROC analysis. Estimated β-coefficients were extracted from the final multivariable regression and points ascribed. Observed and predicted rates of accurate genotype status identification for each possible total score were calculated based on the points assigned. Diagnostic performance including positive and negative predictive values of the weighted score model for predicting genotype-status was assessed based on ROC curve. To evaluate performance of the scoring system, 10,000 bootstrap samples with replacement within each of genotype-positive and genotype-negative MEN1 were created using the same data set. Bootstrap percentile confidence intervals of sensitivity, specificity, PPV, and NPV were obtained.

The strength of the statistical evidence throughout the study was assessed using study statistics (for example, mean/median), measures of uncertainty (IQR), and CIs, together with *P* values in drawing conclusions. *P* < 0.05 was considered statistically significant. Data were analyzed using SAS, version 9.4 (SAS Institute), and R studio.

### Study approval.

Written informed consent was obtained from all patients included in this analysis under the IRB-approved Natural History Study of Parathyroid Disorders (ClinicalTrials.gov Identifier: NCT04969926) conducted at NIH Clinical Center, Bethesda, Maryland, USA.

### Data availability.

Some but not all data are available upon reasonable request to the corresponding author. Data sharing will require evaluation of the request by the local Research Ethics Board and the signing of a data transfer agreement. Values for all data points found in graphs are in the Supporting Data Values file.

## Author contributions

SJ conceived and designed the study with inputs from SKA. CCW, RT, CNU, JMW, LB, CC, AN, SK, SW, SA, LSW, WFS, SKA, JEB, and SJ were involved in the acquisition, analysis, or interpretation of data. Statistical analysis was performed by SA. CCW and SJ drafted the manuscript, which was reviewed by all authors, who take final responsibility for the decision to submit for publication.

## Supplementary Material

Supplemental data

ICMJE disclosure forms

Supporting data values

## Figures and Tables

**Table 1:**
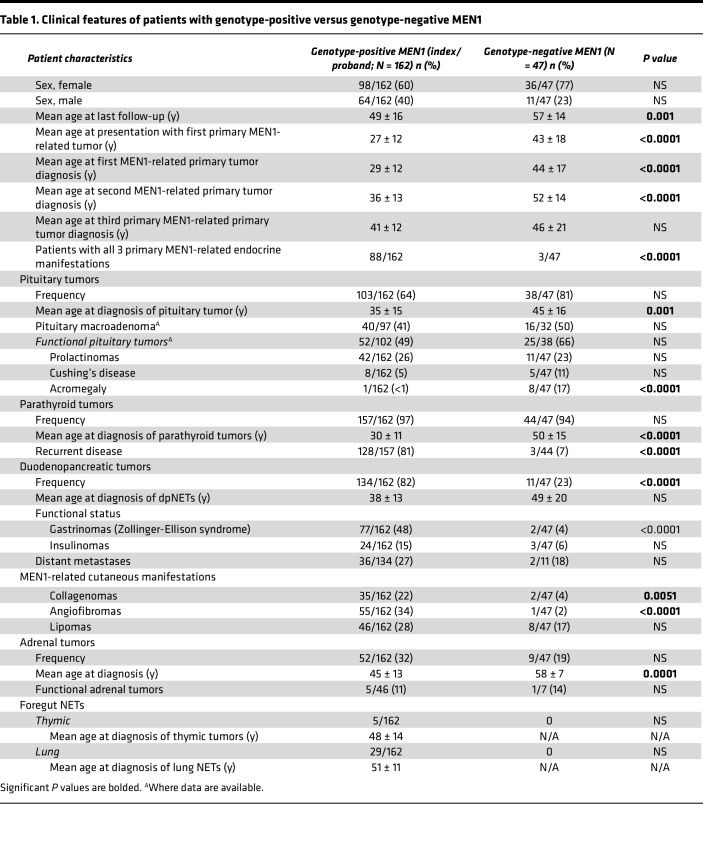
Clinical features of patients with genotype-positive versus genotype-negative MEN1

**Table 2:**
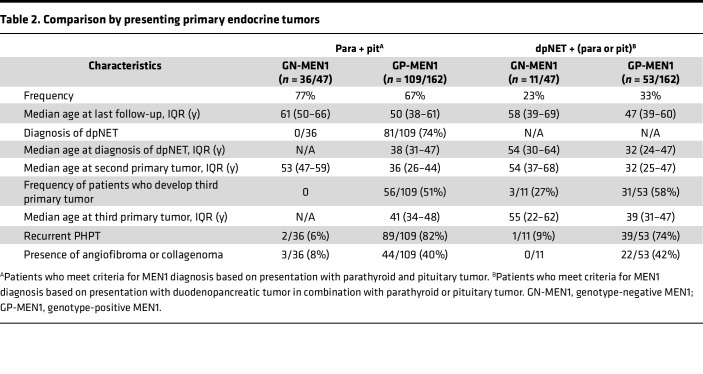
Comparison by presenting primary endocrine tumors

**Table 3:**
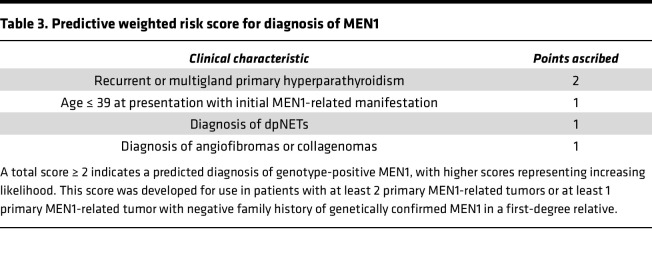
Predictive weighted risk score for diagnosis of MEN1

## Version 1 02/13/2025

In-Press Preview

## Version 2 03/24/2025

Electronic publication
