# Supplementary material for: Genotype-phenotype correlation in multiple endocrine neoplasia type 1
Source: JCI Insight. 2025 Feb 13;10(6):e176993. doi: 10.1172/jci.insight.176993 (PMC11949022; doi:10.1172/jci.insight.176993)
Supplement: Supplemental data [file jciinsight-10-176993-s056.pdf]

Supplementary Appendix

Genotype-Phenotype Correlation in Multiple Endocrine Neoplasia Type 1

Charlita Worthy[1], Rana Tora[1], Chandra Nayan Uttarkar[1], James Welch[1], Lynn Bliss[1], Craig Cochran[1], Anisha Ninan[1], Sheila Kumar[2], Steve Wank[2], Sungyoung Auh[3], Lee S. Weinstein[1], William F. Simonds[1], Sunita K. Agarwal[1], Jenny E. Blau[1], Smita Jha[1]

[1]Metabolic Diseases Branch, National Institute of Diabetes and Digestive and Kidney Diseases, National Institutes of Health, Bethesda, Maryland, USA

[2]Digestive Diseases Branch, National Institute of Diabetes and Digestive and Kidney Diseases, National Institutes of Health, Bethesda, Maryland, USA

[3]Biostatistics Program, National Institute of Diabetes and Digestive and Kidney Diseases, National Institutes of Health, Bethesda, Maryland, USA

Short title: Genetics of MEN1

Clinical Trial number: NCT04969926

Address correspondence to; Smita Jha, M.D., Metabolic Diseases Branch, NIDDK, NIH, Building 10, Room 9C432A, 10 Center Drive, Bethesda, MD 20892. Email: smita.jha@nih.gov; ORCID ID: 0000-0001-9201-3340

Keywords: genotype-negative MEN1, clinical MEN1, diagnostic criteria for MEN1, mosaicism in MEN1, duodenopancreatic tumors in MEN

Supplementary Table 1: Number of variants and variant type in each exon			
**Exon/intron**	**Number of variants**	**Number of index cases**	**Variant type**
2	33	60	Truncating: 21<br>Non-truncating: 12
3	12	19	Truncating: 6<br>Non-truncating: 6
4	10	10	Truncating: 6<br>Non-truncating: 4
5	2	4	Truncating: 2<br>Non-truncating: 0
6	6	6	Truncating: 4<br>Non-truncating: 2
7	10	10	Truncating: 8<br>Non-truncating: 2
8	7	9	Truncating: 4<br>Non-truncating: 3
9	13	15	Truncating: 8<br>Non-truncating: 5
10	13	24	Truncating: 13<br>Non-truncating: 0

Supplementary Table 2: Novel *MEN1* variants or variants of uncertain significance observed in the cohort and their consequences*

DNA change	Protein change	Predicted effect	Phenotype observed at last follow-up
c.43T>C	p.Ser15Pro	not found in gnomAD. WT residue buried in the protein core, mutant residue bigger and more hydrophobic; will cause loss of hydrogen bond formation due to size difference and disturb correct folding.	recurrent PHPT and insulinoma at age 36 (DK-779)
c.108_122dup	p.Leu37_Leu41dup	not found in gnomAD. variant changes the protein coding length.	early onset recurrent PHPT and pituitary adenoma (DK-937)
c.256_259del	p.Ile86ProfsX32	not found in gnomaAD, predicted to cause nonsense mediated decay, located in exon 2 with over 190 pathogenic variants.	recurrent PHPT with initial presentation at age 27, pituitary adenoma and PNET (DK-769).
c.313delC	p.Leu105SerfsX14	not found in gnomAD, predicted to cause nonsense-mediated decay, located in exon 2 with over 190 pathogenic variants.	PHPT at age 26, metastatic PNET at age 32 (DK-358)
c.315delC	p.Tyr106IlefsX13	not found in gnomAD, predicted to cause nonsense-mediated decay, located in exon 2 with over 190 pathogenic variants.	recurrent PHPT and ZES at age 22, pituitary macroadenoma at age 34 (DK-132)
c.334_335dup	p.Ser114ProfsX6	not found in gnomAD, predicted to cause nonsense-mediated decay, located on exon 2 with over 190 pathogenic variants.	recurrent PHPT with initial presentation at age 26, prolactinoma, PNET and thymic carcinoid (DK-986)
c.605del	p.Gly202AlafsX22	not found in gnomAD, predicted to cause nonsense-mediated decay, located in exon 3 with over 90 pathogenic variants	recurrent PHPT with initial presentation at age 27, Cushing's Disease and PNET (DK-143), additional family member with primary MEN1 manifestations.
c.638_639delCCinsAA	p.Ala213Glu	not found in gnomAD, WT residue located on the surface of the protein, mutant residue bigger, less hydrophobic than WT residue which can disturb interactions with other molecules or other parts of the protein	PHPT at age 39 and ZES (DK-977)
c.710C>T	p.Ala237Val	not found in gnomAD, variant residue bigger than WT-residue, variant residue is unlikely to fit which would disturb the local stability of the protein.	recurrent PHPT with initial presentation at age 18, ZES at age 32 (DK-53); additional family member with primary MEN1-related manifestations
c.742del	p.Asp248ThrfsX33	not found in gnomAD, predicted to cause nonsense-mediated decay, located on exon 4 with over 60 pathogenic variants.	pituitary adenoma at age 28 (DK-1296); additional family member with MEN1-related primary manifestation
c.902T>C	p.Leu301Pro	rare in gnomAD, variant residue smaller than WT-residue, predicted to result in empty space in the protein core, WT-residue in a region annotated to form an α-helix, proline disrupts α-helix when not located at one of the first 3 positions of helix, protein structure expected to be severely affected.	recurrent PHPT and PNET (DK-700)
c.982C>G	p.His328Asp	rare in gnomAD	recurrent PHPT and PNET (DK-2236)
c.1089del	p.Glu363AspfsX5	rare in gnomAD, predicted to cause nonsense mediated decay	recurrent PHPT (DK-131)
c.1092_1093del	p.Phe365Ter	not reported in gnomAD, predicted to cause nonsense mediated decay	metastatic PNET (DK-222), additional family members with primary MEN1-related manifestations
c.1102_1104del	p.Ala368del	not found in gnomAD, protein-coding length changes because of in-frame variant and this variant is not located in a repeat region, located in exon 8 with over 90 pathogenic variants.	recurrent PHPT, pituitary adenoma, and PNET (DK-1444); 2 additional family members with primary MEN-1 related manifestations.
c.1152_1162dup	p.Glu388GlyfsX61	not found in gnomAD, prediced to cause nonsense mediated decay, located in exon 8	recurrent PHPT and PNET (DK-1182)
c.1253_1255del	p.Asp418del	not found in gnomAD, protein coding length changes because of this in-frame variant, located in exon 9	recurrent PHPT with initial presentation at age 19, PNET, ZES (DK-1721), 3 additional family members with MEN1 and this variant.
c.1270G>A	p.Glu424Lys	not found in gnomAD, WT residue buried in the protein core, variant residue bigger and positively charged viz. the negatively charged WT-residue - can cause repulsion between residues in the protein core, variant residue unlikely to fit in the correct position and make the same hydrogen bonds or the ionic interactions as the WT-residue	PHPT at age 23, PNET (DK -1290)
c.1399_1400insGA	p.Ala467GlyfsX93	not found in gnomaAD, predicted to cause nonsense mediated decay, located in exon 10 with over 150 pathogenic variants.	recurrent PHPT with initial presentation at age 20, insulinoma at age 25 (DK-273)

*variants were characterized as novel or of uncertain significance based on inadequate evidence of pathogenicity in VarSome and ClinVar in conjunction with published literature (1). Structural effects of missense variants were analyzed using HOPE (2).

**Supplementary Table 3: Genotype-phenotype correlation in genetically confirmed MEN1 by variant type**			
	**Truncating**	**Non-truncating**	**P-value**
Pituitary adenomas			
Frequency	79/119	24/41	NS
Age at diagnosis (years)	33±14	40±17	**NS**
Macroadenomas	32/72	8/25	NS
Functional pituitary adenomas	39/77	13/25	NS
Parathyroid tumors			
Frequency	117/121	40/41	NS
Age at diagnosis (years)	30±11	30±12	**NS**
Recurrent disease	96/117	32/40	NS
Duodenopancreatic neuroendocrine tumors (dpNETS)			
Frequency	99/121	35/41	NS
Presence of insulinomas	17/121	7/41	NS
Presence of gastrinomas	58/121	19/41	NS
Age at dpNET diagnosis (years)	38±13	39±13	NS
Distant metastasis	30/121	6/41	NS
Foregut neuroendocrine tumors			
Thymic NETs	4/121	0/40	NS
Age at diagnosis of thymic NET (years)	50±15	-	
Lung NETs	23/121	6/41	NS
Age at diagnosis of lung NET (years)	51±11	50±6	NS
Adrenal tumors			
Frequency	41/121	11/41	NS
Age at diagnosis (years)	43±12	50±16	**NS**
Dermatologic findings			
Frequency of angiofibromas	44/118	11/40	NS
Frequency of collagenomas	27/118	8/40	NS
Frequency of lipomas	35/119	11/40	NS
NS: not significant			

Supplementary Table 4: Genotype-phenotype correlation in genetically confirmed MEN1 by variant location										
	**Exon 2 (n=60)**	**Exon 3 (n=19)**	**Exon 4 (n=10)**	**Exon 5 (n=4)**	**Exon 6 (n=6)**	**Exon 7 (n=10)**	**Exon 8 (n=8)**	**Exon 9 (n= 15)**	**Exon 10 (n= 24)**	**P-value**
Pituitary adenomas										
Frequency	42	10	5	4	5	5	5	8	16	NS
Age at diagnosis (years)	35±15	42±10	34±15	33±15	21±8	34±16	36±10	33±18	40±17	NS
Macroadenomas	19	3	2	2	4	1	3	2	11	NS
Functional pituitary adenomas	23	4	3	2	3	3	2	4	6	NS
Parathyroid tumors										
Frequency	60	18	9	4	6	10	8	13	24	NS
Age at diagnosis (years)	28±10	34±15	31±12	24±11	26±10	36±13	34±9	30±9	30±10	NS
Recurrent disease	45	16	9	3	4	9	5	11	21	NS
Duodenopancreatic neuroendocrine tumors (dpNETS)										
Frequency	49	18	9	4	4	8	8	10	20	NS
Presence of insulinomas	8	4	1	1	2	2	0	3	2	NS
Presence of gastrinomas	31	9	6	3	2	5	5	5	9	NS
Age at dpNET diagnosis (years)	37±13	40±15	38±14	32±18	36±8	41±9	38±14	38±12	40±13	NS
Distant metastasis	13	1	1	0	2	4	4	0	10	**0.001**
Thymic and lung neuroendocrine tumors										
Lung NETs	12	7	1	0	0	2	1	1	5	NS
Age at diagnosis of lung NET (years)	49±12	53±13	59	-	-	49±6	55	48	52±12	NS
Thymic NETs	2	0	0	0	0	1	1	0	0	NS
Age at diagnosis of thymic NET	55±14	-	-	-	-	32	45	-	-	-
Adrenal tumors										
Frequency	23	9	3	0	2	5	2	2	5	NS
Age at diagnosis (years)	43±15	51±12	47±25	-	43±17	43±10	40±6	42±17	42±7	NS
Dermatological findings										
Frequency of angiofibromas	18	5	3	1	1	2	3	7	11	NS
Frequency of collagenomas	12	0	3	1	2	1	0	3	9	NS
Frequency of lipomas	19	9	4	1	2	1	2	2	4	NS

**Supplementary Table 5: Genotype-phenotype correlation in genetically confirmed MEN1 by central cavity of menin being affected (protein-interacting domain)**			
	**Affected**	**Unaffected**	**P-value**
Pituitary adenomas			
Frequency	62/95	40/63	NS
Age at diagnosis (years)	35 ± 13	35 ± 17	NS
Macroadenomas	25/60	15/36	NS
Functional pituitary adenomas	33/63	19/38	NS
Parathyroid tumors			
Frequency	93/96	62/64	NS
Age at diagnosis (years)	31 ± 12	29±10	NS
Recurrent disease	72/96	54/64	NS
Duodenopancreatic neuroendocrine tumors (dpNETS)			
Frequency	82/96	50/64	NS
Presence of insulinomas	17/96	7/64	NS
Presence of gastrinomas	51/96	26/64	NS
Age at dpNET diagnosis (years)	38 ± 13	38±12	NS
Distant metastasis	21/96	15/64	NS
Foregut neuroendocrine tumors			
Thymic NETs (or thymoma)	4/95	1/64	NS
Age at diagnosis of thymic NET (years)	49 ± 16	45	NS
Lung NETs	19/96	10/64	NS
Age at diagnosis of lung NET (years)	52 ± 11	49 ± 11	NS
Adrenal tumors			
Frequency	36/96	15/64	NS
Age at diagnosis (years)	45 ± 15	49 ± 11	NS
Dermatologic findings			
Frequency of angiofibromas	26/94 (28%)	28/62 (45%)	**0.02**
Frequency of collagenomas	14/94 (15%)	21/62 (34%)	**0.005**
Frequency of lipomas	33/94 (35%)	13/63 (21%)	0.051
NS: not significant			

Supplementary Table 6: Tumor sequencing in patients with genotype-negative MEN1 to detect germline or somatic mosaicism		
**Patient-ID (n=17)**	**Tumor for sequencing* (n=30)**	**Results of MEN1 candidate gene analysis^**
DK-1630	parathyroid + pituitary	two splice site *CDC73* variants (exon-5, c.371-1G>A and exon-17, c.1560-1G>A) in parathyroid tumor but not in corresponding pituitary tumor
DK-1069	parathyroid (2) + pituitary	*MEN1* p.R314Pfs (del28) in one parathyroid gland but not in second parathyroid gland or pituitary tumor
DK-2064	parathyroid + PNET	*MEN1* p.D350Efs*11 and *CDKN2C* p.R68X in parathyroid but not in PNET
DK-79	parathyroid tumors (3)	*MEN1* p.G401Pfs*8 in one parathyroid tumor but not in remaining 2 parathyroid tumors
DK-1318	parathyroid tumors (3)	*CDKN1B* variants p.L70-74del and p.E75X in one parathyroid tumor but not in remaining two
DK-375	parathyroid tumors (3)	*CDC73* p.Q295X and *MEN1* p.R295Q in one parathyroid tumor but not in remaining two
DK-811	parathyroid + pituitary	no *MEN1* variant in any tumor; no variants in any genes shared across tumors
DK-1065	parathyroid + pituitary	no *MEN1* variant in any tumor; no variants in any genes shared across tumors
DK-1396	parathyroid + PNET	no *MEN1* variant in any tumor; no variants in any genes shared across tumors
DK-1024	parathyroid (1)	no *MEN1* variant in tumor
DK-147	parathyroid (1)	no *MEN1* variant in tumor
DK-969	parathyroid (1)	no *MEN1* variant in tumor
DK-394	parathyroid (1)	no *MEN1* variant in tumor
DK-1301	parathyroid (1)	no *MEN1* variant in tumor (multiplex PCR)
DK-1093	parathyroid (1)	no *MEN1* variant in tumor (multiplex PCR)
DK-2127	parathyroid (1)	*MEN1* variants detected (p.Glu195* and p.Phe159fs*26) in tumor but not in germline DNA[#]
DK-2128	parathyroid (1)	no *MEN1* variant in tumor[#]

*parenthesis shows number of tumors analyzed
^two tumors from patients DK-1301and DK-1093 underwent multiplex PCR for *MEN1*. All remaining tumors underwent whole exome sequencing.
[#]see Supplementary Figure 6

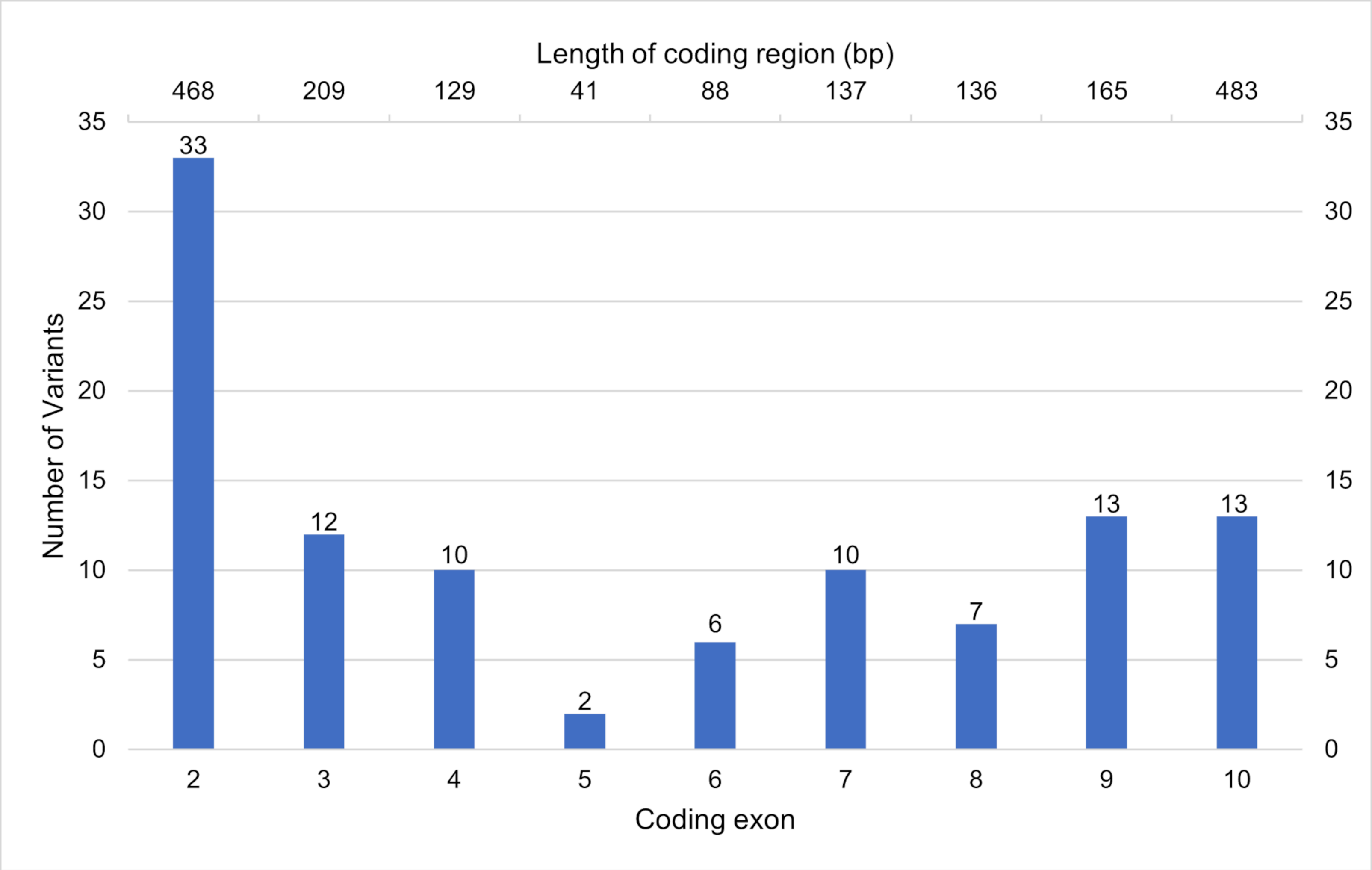

Supplementary Figure 1: Distribution of *MEN1* variants by exons and variant type

Graph showing the relationship to the number of variants and exon size (coding) in *MEN1*

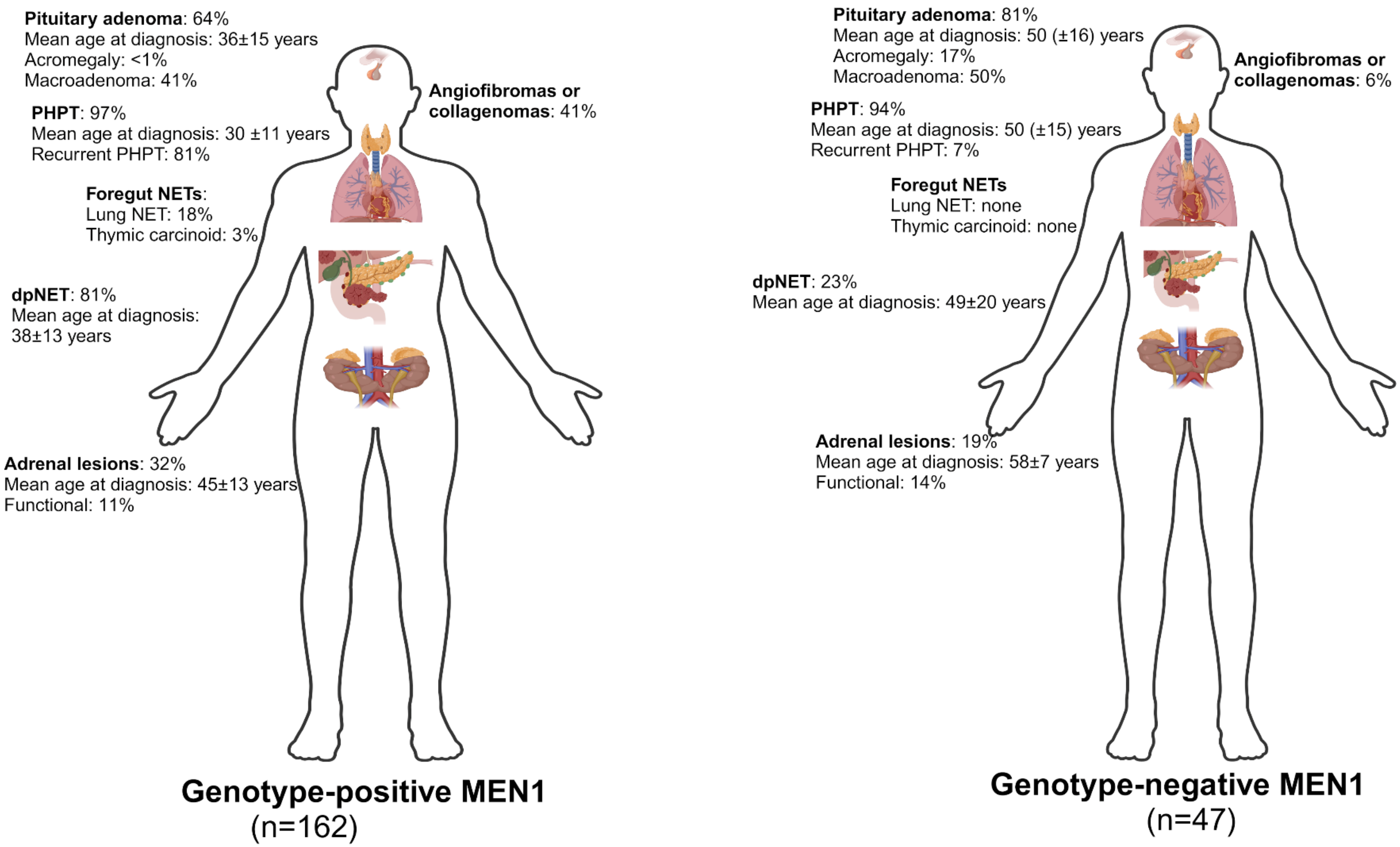

Supplementary Figure 2: Clinical characteristics of genotype-positive *vs.* genotype-negative patients with MEN1

Clinical course of primary MEN1-related endocrine tumors and prevalence of non-endocrine manifestations in study cohort

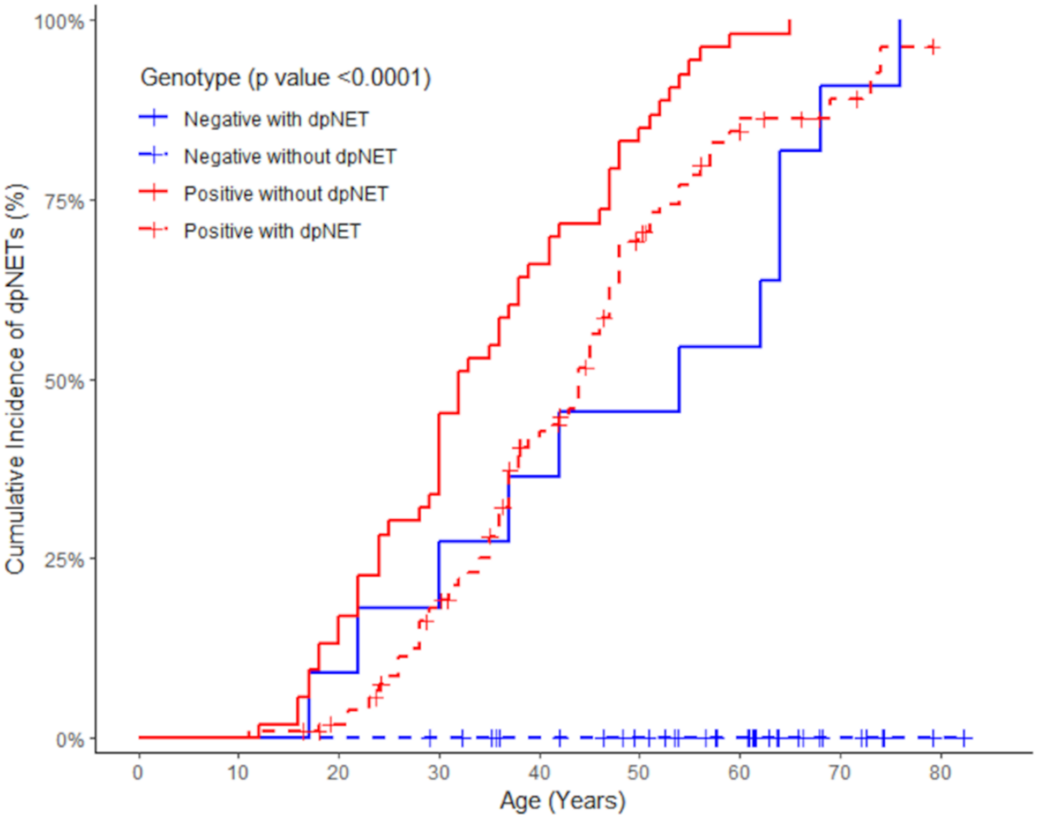

At Risk

Negative with dpNET	11	11	10	9	7	6	5	1	0
Negative without dpNET	36	36	36	35	31	27	20	6	1
Positive with dpNET	53	53	46	35	18	9	1	0	0
Positive without dpNET	109	109	104	84	54	25	9	4	0

Supplementary Figure 3: **Presenting MEN1-related primary endocrine manifestation and genotype-status**

Kaplan-Meir curve relating age at diagnosis on the x-axis to cumulative occurrence of duodenopancreatic neuroendocrine tumors (dpNETs) on the y-axis for both genotype-positive and genotype-negative group of patients. Patients are further stratified within each group based on presenting manifestations at diagnosis of MEN1 (with or without dpNETs). Each step down on the curve refers to a diagnosis of dpNET within the cohort. Crosses refer to age at last follow-up of a patient with no diagnosis of dpNET. The number of individuals in each category at each time point is depicted in the table below the curve. P value denotes statistical comparison for significant differences between any of the four groups. Among patients who presented with dpNETs, there was a significance difference in age at presentation with dpNETs between the genotype-positive *vs.* genotype-negative groups ($P = 0.02$). Among patients who presented with parathyroid ± pituitary tumors (without dpNETs group), there is a significant difference in age-related penetrance of dpNETs with none of the patients in the genotype-negative group developing a dpNET ($P < 0.0001$).

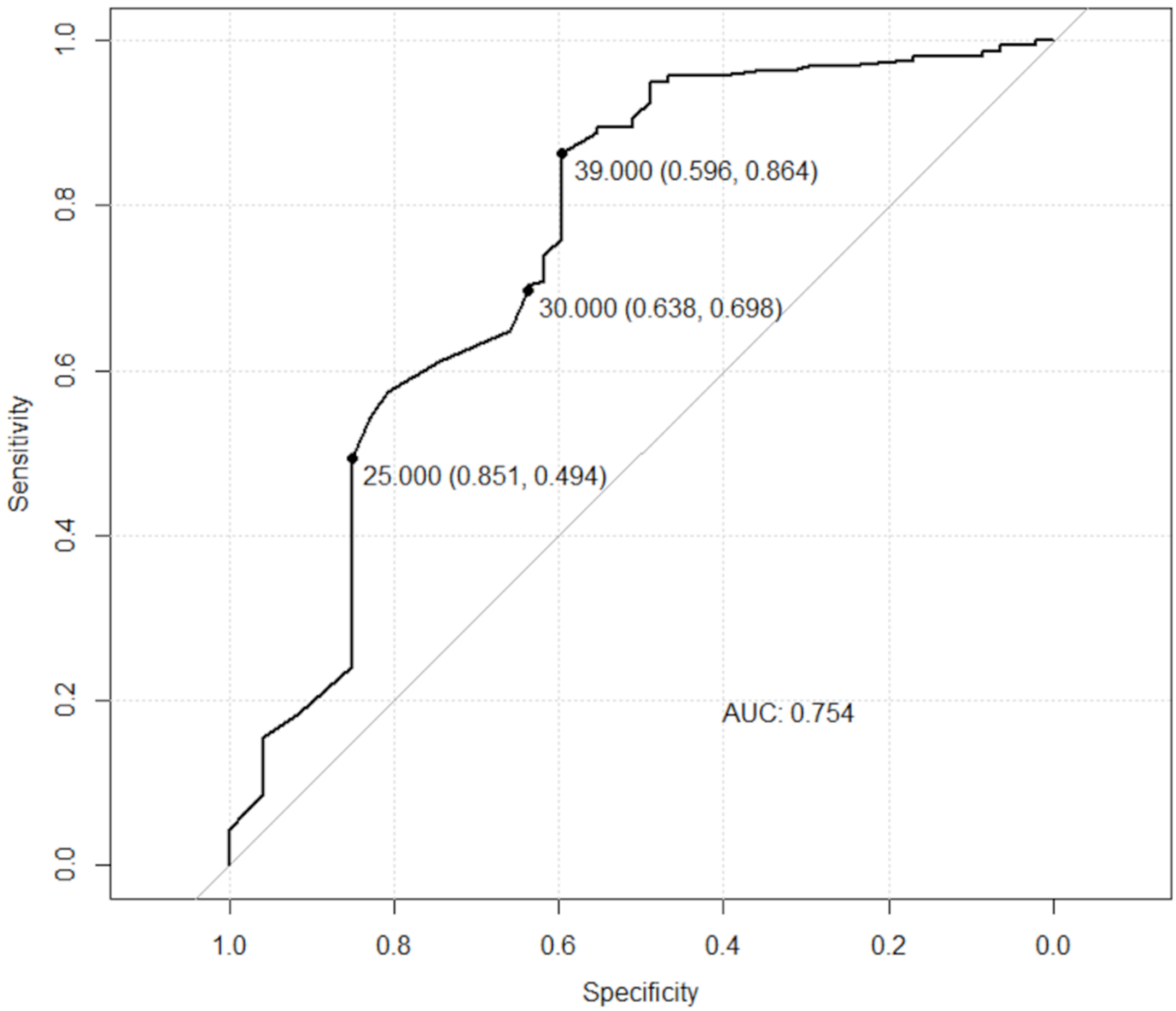

Supplementary Figure 4: Receiver Operating Characteristic (ROC) Curve Analysis

Performance of age of presentation with first MEN1-related primary endocrine tumor in discriminating genotype status in MEN1. ROC curve for age of presentation shows area under curve of 0.75 (95% CI: 0.67-0.84). Cut-off age from left to right provide the smallest distance to perfect distinguishing capability: 25, 30, and 39 years. Of these, age 39 provides the highest sensitivity at 86% with a specificity of 60%.

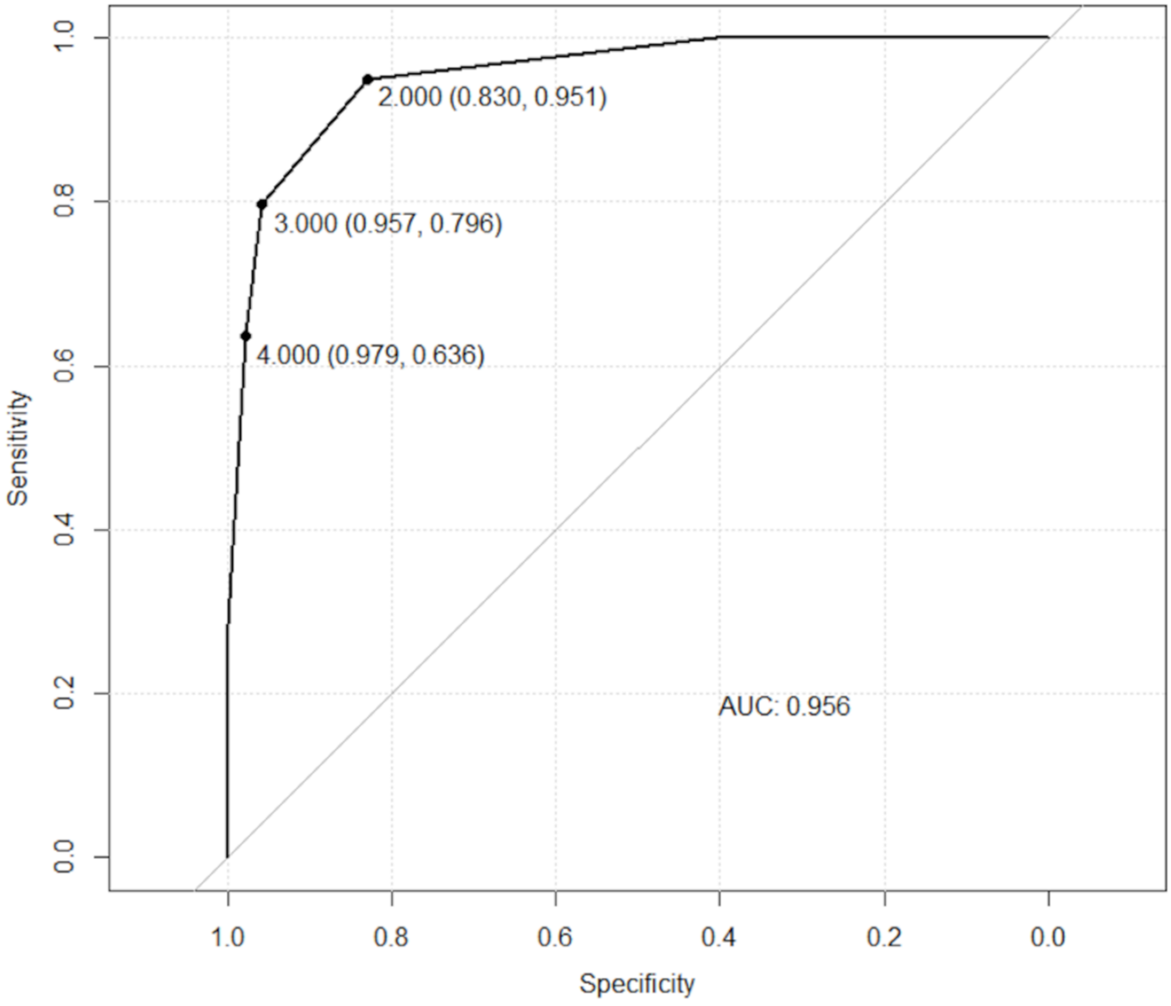

Supplementary Figure 5: Receiver Operating Characteristic (ROC) Curve Analysis

Performance of predictive weighted risk score in discriminating genotype status in MEN1. ROC curve for predictive score shows area under curve of 0.95 (95% CI: 0.92-0.98). Cut-off score from left to right provide the smallest distance to perfect distinguishing capability: 4, 3, and 2 points. Of these, total score of 2 provides the highest sensitivity at 95% with a specificity of 83%.

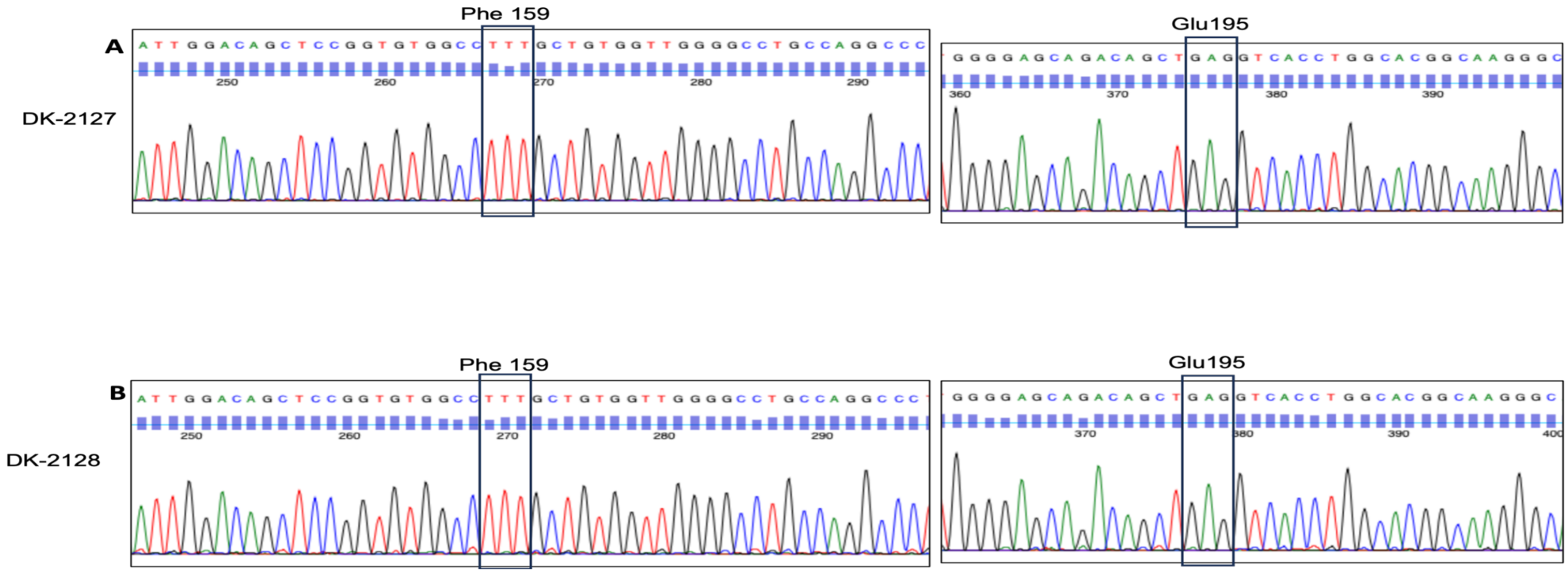

Supplementary Figure 6: Sanger-sequencing of germline DNA from genotype-negative patients

A. Sanger sequencing of germline DNA from Patient DK-2127 with genotype-negative MEN1 and somatic *MEN1* variants indicating absence of variants p.Glu195* and p.Phen159fs*26 identified in parathyroid tumor from the patient. Next generation sequencing was also negative for the variant in germline DNA from the patient. **B.** Sanger sequencing of germline DNA from Patient DK-2128, the sister of patient shown in ‘A’ with genotype-negative MEN1 (primary hyperparathyroidism + positive family history of MEN1 in first degree relative) indicating absence of *MEN1* variants detected in parathyroid tumor of her sister, Patient DK-2127. Next generation sequencing was also negative for the variant in germline DNA from the patient.

References

1. Kopanos C, Tsiolkas V, Kouris A, Chapple CE, Albarca Aguilera M, Meyer R, et al. VarSome: the human genomic variant search engine. *Bioinformatics.* 2019;35(11):1978-80.
2. Venselaar H, Te Beek TA, Kuipers RK, Hekkelman ML, and Vriend G. Protein structure analysis of mutations causing inheritable diseases. An e-Science approach with life scientist friendly interfaces. *BMC Bioinformatics.* 2010;11:548.
